# Variability of Action Potentials Within and Among Cardiac Cell Clusters Derived from Human Embryonic Stem Cells

**DOI:** 10.1038/srep18544

**Published:** 2016-01-05

**Authors:** Renjun Zhu, Michal A. Millrod, Elias T. Zambidis, Leslie Tung

**Affiliations:** 1 Department of Biomedical Engineering, The Johns Hopkins University, Baltimore, MD 21205; 2 Institute for Cell Engineering and Division of Pediatric Oncology, Sidney Kimmel Comprehensive Cancer Center, The Johns Hopkins University, Baltimore, MD 21205.

## Abstract

Electrophysiological variability in cardiomyocytes derived from pluripotent stem cells continues to be an impediment for their scientific and translational applications. We studied the variability of action potentials (APs) recorded from clusters of human embryonic stem cell-derived cardiomyocytes (hESC-CMs) using high-resolution optical mapping. Over 23,000 APs were analyzed through four parameters: APD_30_, APD_80_, triangulation and fractional repolarization. Although measures were taken to reduce variability due to cell culture conditions and rate-dependency of APs, we still observed significant variability in APs among and within the clusters. However, similar APs were found in spatial locations with close proximity, and in some clusters formed distinct regions having different AP characteristics that were reflected as separate peaks in the AP parameter distributions, suggesting multiple electrophysiological phenotypes. Using a recently developed automated method to group cells based on their entire AP shape, we identified distinct regions of different phenotypes within single clusters and common phenotypes across different clusters when separating APs into 2 or 3 subpopulations. The systematic analysis of the heterogeneity and potential phenotypes of large populations of hESC-CMs can be used to evaluate strategies to improve the quality of pluripotent stem cell-derived cardiomyocytes for use in diagnostic and therapeutic applications and in drug screening.

In the last decade, great efforts have been made towards seeking new sources of human cardiomyocytes for various applications, especially for drug cardiotoxicity screening and myocardial repair that require large numbers of cells. Among the candidates, human embryonic stem cells (hESCs) have attracted significant attention, because of their potential to proliferate indefinitely and to differentiate into beating cardiomyocytes (hESC-CMs) *in vitro*. Methods for cardiac differentiation have been evolving rapidly since the work of Kehat *et al*. in 2001, which was based on spontaneous formation of human embryoid bodies (hEBs)^1^. Greatly improved protocols have since been reported, involving the culturing of hEBs in tightly controlled manners (such as forced aggregation, in microwells, etc.), co-culture of hESC colonies with visceral-endoderm-like cells (END-2 line) or use of hESC monolayers^2^. These advances make hESC-CMs a possible candidate for cardiac regeneration^3^ as well as an alternate experimental model for rarely available native human cardiomyocytes. However the interpretation of responses of hESC-CMs to physiological or pharmacological stimuli is still confounded by their immature and heterogeneous state. Attention has been given primarily to their electrophysiological function, using patch clamp, intracellular microelectrodes, and microelectrode arrays as measurement tools. However, currently available characterization is still far from complete (for review, see Blazeski *et al*.^4^).

The action potential (AP) is central to cardiomyocyte function because it not only initiates but also regulates and coordinates tissue contraction. The morphology of APs reflects the net balance among ionic currents across the cell membrane, and is a key signal in excitation-contraction coupling. APs recorded from either single isolated hESC-CMs or cell clusters containing hESC-CMs have widely varying AP morphologies^4^. AP variability can be the result of different culture conditions and cell lines, but independent of that, is reflective of the subtype and maturity of these *in vitro* generated cardiomyocytes^5^,^6^,^7^. Among different laboratories, APs recorded from hESC-CMs have generally been classified as one of three subtypes: nodal-like, atrial-like or ventricular-like^8^,^9^,^10^,^11^,^12^,^13^,^14^,^15^,^16^,^17^,^18^ corresponding to the major CM phenotypes in adult myocardium. However, the invasiveness and time-consuming nature of direct electrophysiological recordings substantially limit the sample sizes of these studies (ranging from 15–125 in the cited studies, with an average of 50 samples) making it unclear whether predominant phenotypes are still present in larger, more representative cell populations.

Previously, we^19^,^20^ and others^21^,^22^,^23^ showed that optical mapping can be used to investigate the electrophysiology of confluent populations of hESC-CM. Combined with a high resolution imaging system, it is practical to study cells in large populations all at once. Following our previous observation that APs recorded from beating areas of hEBs (which are dissected out and which we will refer to as cardiac cell clusters) from the same differentiation batch had a broad variation in morphology across clusters^4^, we obtained a large dataset of APs of hESC-CM populations within cardiac cell clusters in this study, and focused on characterizing the variability and identifying the presence of predominant phenotypes. We used well-established parameters such as spontaneous activity and AP duration (APD), as well as novel waveform-based analysis methods to characterize the variability among and within cardiac cell clusters. These measurements represent the first systematic analysis of the variability and presence of phenotypes within a large cell population. We anticipate that this approach can also be used to evaluate new strategies designed to reduce the phenotypic variation within hESC-CM populations and improve their quality for use in diagnostic and therapeutic applications and in drug screening.

## Results

### Spontaneous and electrically stimulated activity of cardiac cell clusters

**Figure 1:**
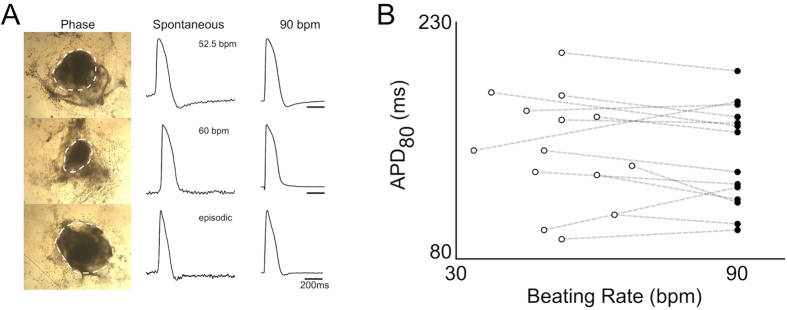
Spontaneous activity of cardiac cell clusters. (**A**) Left column: three beating hEBs at 14 days after initiating cardiac differentiation. Dashed contours indicate beating areas. Middle column: spontaneous action potentials recorded from a site in each of the cardiac cell clusters derived from the three hEBs. Right column: action potentials recorded from the same sites of each during 90 bpm pacing. (**B**) APD_80_ of spontaneous and paced cardiac cell clusters. Open circles: APD_80_ of spontaneous APs recorded from 14 cardiac cell clusters. Closed circles: APD_80_ of APs recorded at fixed 90 bpm pacing rate. Dashed line connecting open and closed circles indicates the same cluster.

We started to see spontaneously beating hEBs around day 10 of differentiation. The number of beating hEBs varied as differentiation proceeded and also varied among differentiation batches. The clusters used for this study were obtained from a single batch of differentiation where more than 90% of hEBs were beating by day 15 (day of mechanical dissection). Although similar numbers of undifferentiated hESCs were seeded for hEB formation (5000 cells/hEB), clear differences in size and shape of hEBs and their beating areas were observed (Fig. 1A, left column). After mechanical dissection, all cardiac cell clusters (beating areas of hEBs) attached to the coverslip and recovered spontaneous beating within 5 days, prior to being optically mapped.

From the 55 clusters obtained from the batch, spontaneous APs were recorded using optical mapping. Both continuous (35 clusters) and episodic (20 clusters) patterns of beating were observed, the latter being identified by the existence of at least 4 seconds of quiescence between APs during the recording. Among continuously beating clusters, beating rate was unstable in 6 clusters. Action potentials recorded from different clusters exhibited different spontaneous rates and had clearly different morphologies (Fig. 1A, middle column). The average beating rate of stable, continuously beating clusters was 62 ± 21 bpm (mean ± SD), and their average APD_80_ (action potential duration at 80% repolarization) was 165 ± 49 ms (n = 29). Because action potential characteristics and their underlying ionic currents are known to be rate-dependent, we tested whether some of the variability in APD_80_ of the clusters could be secondary to the differences in the beat rates. Thus, electrical pacing was applied with a pair of platinum electrodes. In the examples shown, a fixed rate of 90 bpm was able to pace-capture, and reduced, but did not eliminate the differences among APs from different clusters (Fig. 1A, right column).

Overall, 14 of the 29 continuously beating clusters with stable beating rate could be entrained by electrical stimulation at 90 bpm. For these 14 clusters, individual APD_80_s measured during spontaneous beating did not correlate with the beat rate (Fig. 1B, open circles), and different APD_80_s were observed even among clusters with similar beat rate. When 90 bpm pacing was applied, there was no significant change in the mean or SD of APD_80_ (147 ± 36 ms without pacing compared with 143 ± 33 ms with pacing, Fig. 1B). In most cases, the increase in beating rate from spontaneous rate to 90 bpm produced a drop in APD_80_, consistent with the negative rate-dependence observed in natural human cardiomyocytes/tissue, although it was the opposite case for 4 of the 14 clusters where APD_80_ increased instead. The large variability in APD_80_ at 90 bpm and the distinctly different rate-dependences among clusters indicate that both rate-independent and rate-dependent effects contribute to the variable AP durations observed at spontaneous beat rates.

### Variability of APs within cardiac cell clusters

**Figure 2:**
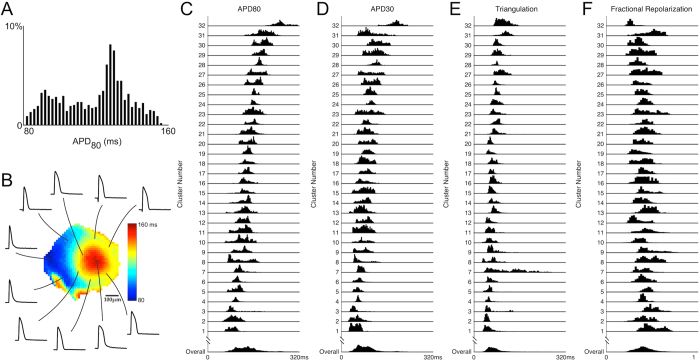
Variability of action potentials within a single cardiac cell cluster and among different clusters. (**A**) APD_80_ histogram of action potentials recorded from a cardiac cell cluster (cluster 9). Two peaks in the histogram indicate subpopulations with predominantly long or short durations. (**B**) Sample action potentials recorded from this cluster. Center: APD_80_ map, showing two distinct regions with long or short AP durations. (**C–F**) Single AP parameter histograms (APD_80_, APD_30_, triangulation and fractional repolarization; see main text for definition) of 32 cardiac cell clusters paced at 90 bpm. Each row represents one cluster. The overall distribution (bottom row) is the summation of all recording sites (n = 23117) from these 32 clusters.

We first studied rate-independent variability of APs within cardiac cell clusters by pacing the clusters at a common rate of 90 bpm, and then recording the spatial distribution of action potentials via high-resolution optical mapping. A representative example is shown in Fig. 2A, where APD_80_ of 981 recordings obtained from a single cluster are shown as a histogram. The average APD_80_ was 119 ± 19 ms, and two prominent distribution peaks were observed in the histogram, suggesting the existence of two subpopulations within the cluster. The advantage of optical mapping is its ability to discriminate whether the two subpopulations are interspersed or whether they cluster together spatially. In the spatial map of this cluster (Fig. 2B), two distinct regions having either long or short APD_80_ were apparent, indicating that on a local scale of the order of a hundred microns, cells have similar AP duration, but on a larger scale of the order of a mm, different AP durations can emerge.

We then compared AP variability within each cardiac cell cluster to that among the entire cluster population. Fixed 90 bpm recordings were obtained from 32 cardiac cell clusters with an average of 722 ± 252 recording sites in each cluster. The average and variation in AP parameters are summarized in Table 1. Even though some of these clusters had spontaneous activities (including the 14 continuously beating clusters analyzed in Fig. 1) while others were quiescent, we did not observe systematic differences in APs when they were paced at 90 bmp that can be correlated to their spontaneous activities. Single AP parameter distributions for individual clusters were clearly different (Fig. 2C–F). In some clusters (e.g, clusters 4, 24, 25, 28), the distributions of all AP parameters were relatively narrow, indicating a relatively homogeneous population of cells within the cluster, whereas in other clusters (e.g. clusters 8, 21, 27, 30) AP parameters were distributed over a wide range and had multiple peaks, revealing multiple sub-populations or a spectrum of cells. In other clusters (e.g. cluster 7), while distributions for some AP parameters (APD_80_, APD_30_ and fractional repolarization) were relatively narrow, much wider distributions were found in triangulation. Thus, AP variability presents differently for different AP parameters. Therefore differences in subpopulation identification may be expected when a single AP parameter (such as APD_80_) is used compared to when multiple AP parameters, or the entire AP waveform is used.

**Figure 3:**
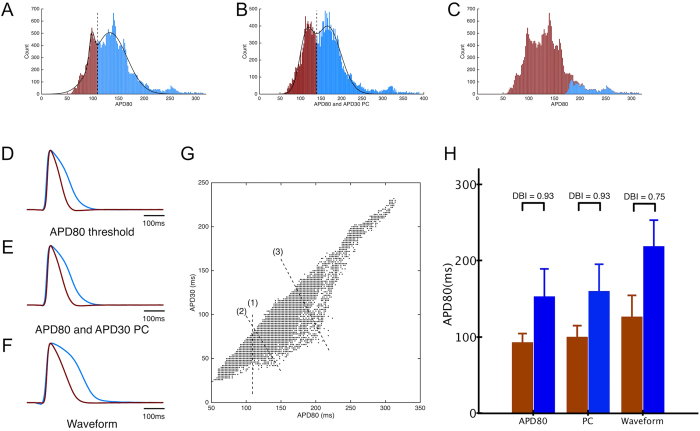
(**A**) Histogram of APD_80_ from all recording sites from 32 clusters. Solid lines show fits of the data by two Gaussian curves, and dashed line indicates threshold used to divide groups of APs (shown in brown and blue). (**B**) Histogram of the principal component (PC), which is a linear combination of APD_80_ and APD_30_. Solid lines show fits of the PC data by two Gaussian distributions, and dashed line indicates threshold used to divide groups of APs (shown in brown and blue). (**C**) Histogram of APD_80_ with waveform-based grouping, with colors showing different groups. Stacking of different colors indicate overlap in APD_80_ parameters between groups. (**D–F**) Average AP traces obtained by grouping APs based on APD_80_ threshold, PC of APD_30_ and APD_80_, and waveform analysis, respectively. (**G**) Scatter plot of APD_80_ and APD_30_ for all recorded APs. Dashed lines represent linear classification boundaries according to (1) threshold using APD_80_, (2) threshold using the principal component, which is a linear combination of APD_30_ and APD_80_ and (3) waveform analysis. (**H**) Comparison of APD_80_ between groups for the three different grouping methods. Colored bars correspond to groups of APs shown in (**A–C**). Waveform analysis resulted in groups with the most distinct AP differences, as indicated by the lowest DBI value.

To further investigate the potential subpopulations among these cardiac cell clusters, we applied and compared different grouping strategies. Starting with APD_80_, we fitted two Gaussian distributions to the overall distribution of the entire population, and used the crossover value as a threshold that divided APs into two groups, namely groups of long or short AP, respectively (Fig. 3A). The average action potentials from the two groups are shown in Fig. 3D. We then added another action potential parameter, APD_30_ to our analysis. Using principal component (PC) analysis, we identified the linear combination of APD_80_ and APD_30_ that accounted for most of the variability in the population, and its distribution is shown in Fig. 3B. By fitting two Gaussian distributions and using the crossover value, we again divided APs into two groups, and the average AP from each group is shown in Fig. 3E. In a third approach, we used an automated unsupervised algorithm to provide an unbiased grouping of APs based on their wave shapes across the entire cluster population. Figure 3C shows the grouping results displayed in terms of APD_80_ and color-coded by the group assignment. Only a small fraction of the action potentials (in blue) were labeled as different from the bulk of the population, leading to a very different result than those using AP parameters alone (Fig. 3A,B). The average APs from the two groups identified by waveform analysis are shown in Fig. 3F. Compared with the average APs using AP parameter analysis (Fig. 3D,E), these had greater differences in morphology (Fig. 3F), with one group having a shorter AP with more triangular profile, and the other group having a longer AP and a longer plateau phase. Differences among the three grouping methods can be visualized in the joint distribution of measured APD_30_ and APD_80_ (Fig. 3G). Here, dashed lines have been drawn corresponding to decision boundaries between the two groups determined by the analysis of: (1) APD_80_ only, (2) APD_80_ + APD_30_ PC, and (3) entire waveform. We found that while the APD_80_-only method always grouped cells with same APD_80_ values together, the variability in their APD_30_ (as indicated by the vertical spread in the joint distribution) resulted in APs groups that could include very different AP shapes. The inclusion of APD_30_ introduced a tilt to the threshold line (compare lines 1 and 2), indicating the contribution of both parameters to the subtype identification. To quantify the distinction between groups, we used the Davies-Bouldin Index (DBI, Fig. 3H), which compares within-group tightness against between-group separations, such that lower DBI indicates better clustering (tighter groups and/or larger separations). DBI was lower for groups identified by waveform analysis (0.75) than when groups were identified by APD_80_-threshold (0.93) or PCA (0.93) (Fig. 3H).

**Figure 4:**
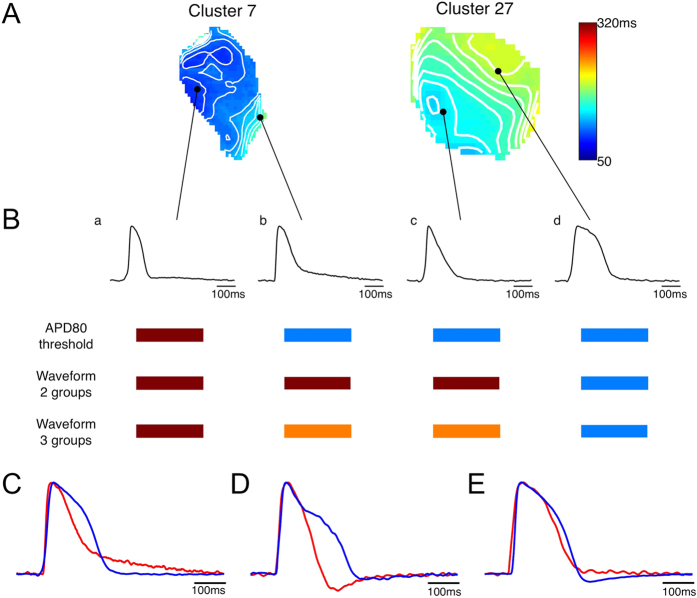
Comparison between grouping by single AP parameter (APD_80_) and by waveform analysis. (**A**) APD_80_ map of two clusters. (**B**) APs recorded from locations indicated by black dots in (**A**), showing significant differences in AP morphology. Grouping results using different methods are shown as color-coded bars below. APs having the same APD_80_ (**C**) or APD_30_ (**D**) can have very different morphology. (**E**) APs having both same APD_80_ and APD_30_ have similar shapes.

Differences in the outcomes of the parameter and waveform analyses are especially apparent in comparing APs within individual clusters (e.g, clusters 7 and 27 in Fig. 4). APD_80_ maps (Fig. 4A) showed considerable variability between the two clusters, reflecting differences among the AP waveforms (Fig. 4B). Among the 4 APs shown, one had short duration with rapid repolarization (a), one had a short duration with a slow repolarization tail at the end of the AP (b), one was very triangular (c) and the last had a long duration with rapid repolarization (d). Different groupings were obtained with the single parameter analysis (here, being APD_80_) compared with the waveform analysis. APD_80_ analysis grouped APs b and c with AP d, whereas waveform analysis grouped these APs with AP a. The limitations of single parameter analysis are also illustrated for APs having the same APD_80_ (Fig. 4C) but drastically different morphology. Analysis in terms of other parameters (such as APD_30_ in Fig. 4D) also encounter morphological differences. Only when both APD_80_ and APD_30_ were used together, were the morphological differences greatly reduced (Fig. 4E). Waveform analysis was successful in separating these APs into different groups. Furthermore, the slower repolarization and more triangulated shapes in APs b-c could be captured by analyzing all of the APs as 3 groups, whereupon APs b-c were identified as belonging to the third group.

**Figure 5:**
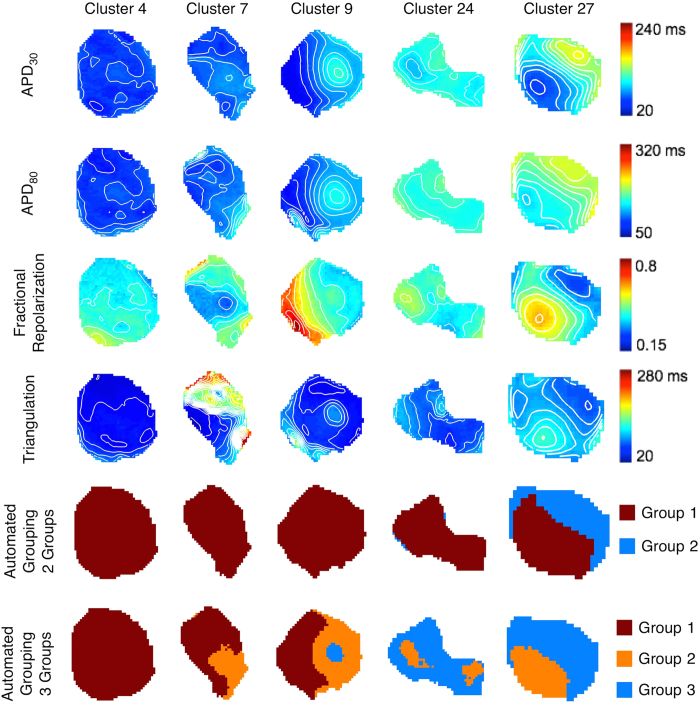
Spatial maps of different AP parameters or groups for five different cell clusters. Top four rows: Spatial distributions of APD_30_, APD_80_, fractional repolarization, and triangulation in the clusters. Iso-parameter contours are 10 ms apart for APD_30_, APD_80_ and triangulation and 0.05 apart for fractional repolarization. Maps in the same row share same color bar. Bottom two rows: Grouping resulting from the automated algorithm and assumption of 2 (second to last row) or 3 (last row) groups. Colors signify different groups assigned by the algorithm. Columns: clusters 4, 7, 9, 24 and 27.

We then expanded our analysis of spatial heterogeneity to two other metrics of AP shape: triangulation and fractional repolarization. Five representative clusters are shown in Fig. 5 (one cluster per column). In some cases (cluster 4), APD_30_, APD_80_, fractional repolarization and triangulation were fairly uniform across the cluster, indicating a single population of cells. In other cases (clusters 9, 27) the 4 AP parameters had similar patterns showing two distinct regions (e.g, on the left and right sides of cluster 9, and lower left and upper right sides of cluster 27) indicative of two subpopulations, although for cluster 27 the regions were not entirely concordant across parameters. In still other cases (cluster 7, 24) the patterns for the AP parameters were dissimilar. By using the automated algorithm with entire AP waveform, cells among the clusters were separated into 2 groups, with spatial patterns as shown (Fig. 5, second to last row). The 2-group analysis revealed that 14 of 32 clusters contained cells from different groups. This coexistence of cells from different groups is more notable with automated 3-group analysis (Fig. 5, last row), where 17 clusters had cells from all 3 groups, 12 clusters had cells from 2 different groups, and only 3 clusters had cells from a single (and different) group. Furthermore, cells belonging to different groups were not intermingled and tended to occupy separate areas within a cluster (Fig. 5, last two rows) even though spatial information was not incorporated into the grouping algorithm. Separate groups could be identified even in clusters where the patterns of APD parameters were discordant (e.g. cluster 24). Additionally, our analysis resulted in the identification of individual AP phenotypes that were common to multiple clusters (e.g., group 3 in clusters 9, 24, and 27). Note, however, that the increased number of groups was accompanied by a higher DBI value (0.91 for 3 groups vs. 0.75 for 2 groups), suggesting that 2 groups better describe this particular population of action potentials.

**Figure 6:**
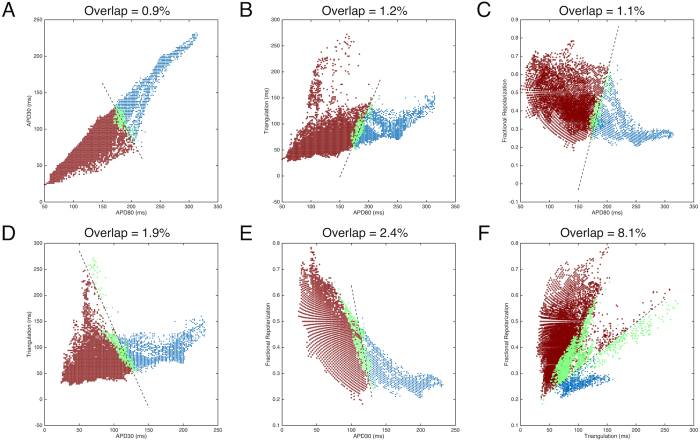
Pairwise scatter plots of individual AP parameters for all recorded APs, color-coded by grouping results defined by waveform analysis. Dashed lines indicate linear decision boundaries separating the two groups in the parameter space, derived by support vector machines (SVMs). APs of each group falling on the opposite side of the boundary (the overlaps, highlighted in green) were quantified as percentage of the total APs and are given above each panel.

For visualization and discussion purposes, we plotted all APs as scatter plots of joint distributions of pairs of AP parameters, along with their group labels by waveform analysis in colors (Fig. 6). Although paced at the same rate, the entire set of APs encompassed a large area in all 2D spaces of AP parameter pairs, indicating a high degree of variability. When APs were divided into two groups, they occupied relatively distinct regions in all 2D spaces. Linear boundaries were determined by support vector machines (SVMs) within each 2D space, and shown as dashed lines. The degree of overlap of the two groups was defined as the total percentage of APs of each group located outside the boundary of their respective groups, and ranged from 0.9% to 8.1% across all of the 2D spaces, indicating the influence of features of AP shape not captured by just a pair of AP parameters. In general, the overlap in the 2D spaces with AP duration parameters (Fig. 6A) was smaller than that in 2D spaces with triangulation and fractional repolarization parameters (Fig. 6F), suggesting that AP duration parameters have a stronger contribution in grouping than triangulation or fractional repolarization.

### Rate dependency

**Figure 7:**
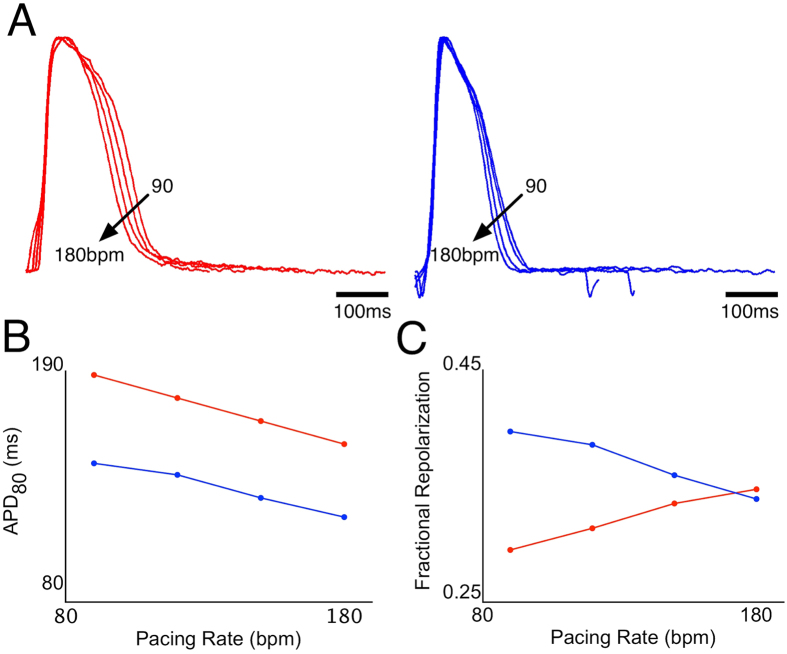
Rate dependence of action potentials in a cell cluster (cluster 26). (**A**) Action potentials recorded from two different locations in the cluster having long (left, red traces) and short (right, blue traces) APs, at 90, 120, 150 and 180 bpm pacing. (**B**) Rate dependence of APD_80_ at the two recording sites. (**C**) Rate-dependence of fractional repolarization at the two recording sites.

Depending on the cluster, electrical pacing could stimulate APs up to 330 bpm while maintaining 1:1 capture, allowing us to study the rate-dependence of paced APs (Fig. 1B). We hypothesized that rate-dependency could be an additional means by which different sub-populations (phenotypes) of cells could be discriminated. In a cluster having two regions with different AP durations (cluster 26), electrical pacing was applied from 90 to 180 bpm in 30 bpm increments. While all recording sites (n = 585) in this cluster had rate-dependent shortening of APD_80_, the rate dependencies of fractional repolarization varied greatly, being positive (increased fractional repolarization with increased rate) in some regions (n = 180) and negative in others (n = 405). Two representative recordings from this cluster and their corresponding APD_80_ and fractional repolarization rate-dependencies are shown in Fig. 7. The differences in rate-dependencies suggest that the use of multiple pacing rates may further assist in identifying phenotypic differences.

## Discussion

Previous studies have shown that APs generated by hESC-CMs are heterogeneous in their electrophysiological properties and not fully like adult phenotypes^4^,^6^,^24^. A fundamental question is: down to what structural level does this phenotypic heterogeneity extend? Prior studies using microelectrode impalements of hEBs showed that APs differ substantially among different hEBs but were mostly similar within individual hEBs^9^ or within cell clusters^12^. Our results show that APs vary over a wide range across hEBs as well as over a smaller, but still substantial range within hEBs. Although numerous research groups have reported percentages of nodal-like, atrial-like and ventricular-like cells at a stage of differentiation similar to ours (20–24 days)^10^,^14^,^15^,^25^, our results demonstrate that different phenotypes are difficult to distinguish in at least two ways: 1) individual parameters are insufficient to divide populations of APs into different groups, since APs with similar parameter values (such as APD_80_) could have very different morphology indicative of different phenotypes, and 2) the wide range and continuous variability of APs make it extremely difficult to subjectively determine cut-offs between groups. By using an automated grouping algorithm that analyzed entire AP waveform shapes, we able to objectively sort the data into 2 or 3 maximally separated groups.

Cardiac cell clusters derived from hEBs are self-assembled, three-dimensional aggregates of cells. In contrast to flat cultures on two-dimensional substrates, the three-dimensional organization of the cells provides a natural microenvironment for the cells, allowing them to form and remodel their surrounding extracellular matrix, to undergo paracrine and autocrine signaling, and to contract against a compliant mechanical load, while maintaining independence among clusters. To reduce variability due to culture conditions, we used a highly efficient differentiation protocol^20^,^26^ to obtain large numbers of beating hEBs sufficient for variability studies from the same differentiation batch. Beating cell clusters were mapped within a narrow time window, 21–24 days after initiation of differentiation. Fixed-rate pacing was used so that APs could be compared at the same beat rate. As a caveat, there still remained several uncontrolled experimental variables. Although similar numbers of hESCs were seeded to form hEBs, the ensuing hEBs still had different sizes, shapes and beat rates, which can influence the differentiation process. In addition, the mechanically dissected cardiac cell clusters may only represent a subset of all cardiomyocytes. Also, the uncontrolled presence of nonmyocytes in the cell aggregates can influence the maturation of the embedded hESC-CMs^27^, although methods to eliminate nonmyocytes have been reported^18^,^28^. Our differentiation method also did not target a specific cardiomyocyte phenotype, although such strategies have been reported for the ventricular phenotype, where relatively uniform, ventricular-like action potentials have been observed^29^.

Sharp microelectrodes and multiple impalements have been used to study the variability in electrophysiology of hEBs^9^,^12^, but in practice are limited to around 10 or less per hEB. In contrast, high-resolution optical mapping provides hundreds of recordings from precise locations within single cardiac clusters without mechanical disruption or cellular injury due to impalements. However, there are limits on true spatial resolution, owing to diffuse light scattering of the fluorescent signal and volume integration of signals on the recording path^30^,^31^. Electrical coupling among neighboring cells can also electrically average APs within close proximity. The boxcar filter applied in our data analysis, which was necessary to improve signal quality, also limits the spatial resolution in our method. Nonetheless, significant heterogeneity in AP parameters was always observed, indicative of a genuine variation in electrophysiological phenotypes. This finding is not unexpected in hEBs during early stage differentiation, as they are thought to mimic human embryo development^9^,^32^ and have the potential to develop into all major phenotypes of adult cardiomyocytes. Another limitation of optical measurements is that they measure only relative changes in transmembrane voltage and not absolute values (unless advanced methods such as dual wavelength measurements and microelectrode calibration are used)^33^,^34^. Nevertheless, they have fast responses that can faithfully track the transmembrane voltage and preserve AP shape^35^,^36^.

One possible confounding effect in our experiments is that electrotonic influences among cells can produce gradients in action potential duration even in a homogenous cell population. This is particularly evident when action potentials propagate away from a stimulus site, in which case those nearer the site have longer durations than those farther away^37^. These effects are also modulated by tissue dimension, tissue boundaries and structural heterogeneity^38^,^39^,^40^. We do not believe that our results are greatly influenced by such effects. The spatial dimensions of our EBs are much smaller than the hearts in which such effects have been observed, restricting the amount of repolarization gradients that can develop. The differences in APDs observed in previous studies of electrotonic effects are of the order of 10–20 ms, much smaller than the differences we observed within and among clusters (~100 ms). The activation times across our clusters were typically less than 15 ms, and APD shortening did not correlate with the longer activation times. The short activation times, and smooth activation sequences across clusters, also ruled out conduction delays secondary to structural heterogeneities that could produce APD gradients. In addition, variation in APD were observed across the entire cluster, without sharp gradients along the edges where the boundary effects might occur, and significant AP variability occurred among clusters, which is independent of electrotonic effects.

Our high-resolution measurements of early differentiated clusters suggest that a relatively broad distribution of APs is present within the clusters (Fig. 2), although not as broad as across the entire set of 32 clusters that was studied. In many, but not all clusters, a histogram of APD_80_ that was either narrow or else wide with multiple peaks was observed, suggestive of one or multiple predominant phenotypes. APD_30_, triangulation and fractional repolarization are other ways to quantify the plateau and repolarization phases of the AP, which in turn are manifestations of the underlying ionic currents which have differing temporal and voltage-dependent kinetics^41^. APD_30_ is a rough measure of plateau duration, while APD_80_ is a measure of the combined plateau and repolarization durations. Triangulation^42^ is roughly the duration of phase 3 repolarization, and is considered to be proarrhythmic when prolonged. Fractional repolarization is the fraction of the total APD involved with late rapid repolarization. This parameter is also akin to the comparison between plateau (duration between APD_30_ and APD_40_) and repolarization (duration between APD_70_ and APD_80_) phases defined by others to distinguish between ventricular-like cells and atrial-like cells^43^. Our results showed that the spatial patterns of groupings based on each of the four parameters were usually but not always concordant with one another (Fig. 5), raising the question of which parameter(s) is most important.

Ideally, molecular markers would be used to identify subtypes of hESC-CMs, but at present, these are limited primarily to MLC2v or IRX-4 for the ventricular subtype^29^,^44^,^45^ and cannot be used on live cells. Classification by AP morphology is still the most widely adopted method by which cardiomyocyte phenotypes have been assessed by microelectrode or optical recordings^14^,^15^,^16^,^17^,^18^,^22^,^23^, Major subtypes of hESC-CMs (nodal-like, atrial-like, ventricular-like) have most often been identified subjectively by the similarity of their AP shapes to those of the adult phenotypes in terms of AP parameters. Only a few studies specified quantitative criteria for classification, including APD_90_^13^, combination of APD_90_ and beat rate^12^, ratio of APDs^43^,^46^, and a combination of AP amplitude, upstroke velocity, APD_50_ and APD_90_^14^,^25^. In this study, we showed that grouping by histogram analysis of single parameters does not assure consistent AP morphology; action potentials having the same APD_80_ or APD_30_ can have differing AP shapes reflecting different phenotypes (Fig. 4C,D). Increased numbers of parameters result in improved grouping, with the ultimate representation being the entire waveshape.

Analysis of the entire data set of APs (recorded across 32 hEBs) by an automated machine learning algorithm^47^ divided the APs into different groups based on their shape and similarity to one another. This method also produced the most distinctive differences in AP waveforms between groups (Fig. 3F). Within individual hEBs the groups occupied separate spatial regions rather than being intermixed, even though the automated algorithm did not incorporate spatial information. This finding suggests that physical location or cell-cell interactions can influence phenotypic specification during hESC-CM differentiation, which is manifested in the patterns of individual parameters. Our use of the entire set of APs across all clusters also enabled cells that belong to same group to be identified in multiple clusters (Fig. 5), which would not be possible by separate analyses of individual clusters. It should be noted that although APs were divided into 3 groups, those groups do not necessarily correspond to nodal-, atrial- and ventricular-like cells, but perhaps, to precursor or intermediate stage cells. Further studies are warranted to develop more advanced methods for AP shape analysis and ultimately, phenotypic classification.

A further improvement for the discrimination of different phenotypes, which has not been employed so far by other laboratories, is the use of different pacing rates, which takes advantage of the different rate-dependent kinetic properties of the ionic currents underlying the AP. As we showed in one example, with increasing rate APD_80_ decreases all across the cluster, whereas fractional repolarization may change in opposite directions in different regions of the cluster (Fig. 7).

In terms of future applications, we have presented a strategy to obtain large datasets of APs via optical mapping and to analyze them for their electrophysiological variability and grouping. We envision that this approach will have broad applications as a tool in the study of stem cell-derived cardiomyocytes in the context of differentiation^2^, maturation^24^, production^48^, drug screening^49^, disease modeling^50^ and myocardial regeneration^51^. Some examples of questions that could be addressed include: does the monolayer method of differentiation, where paracrine and juxtacrine interactions among the cells is less than in the hEB, result in a more diverse or more homogeneous distribution of cell phenotypes? When do and how many predominant phenotypes emerge during time in culture? Do certain cell lines, culture conditions or bioengineering approaches to improve maturation favor the rapid emergence of a common phenotype? How varied are responses of the cell population to drugs that affect repolarization? What is the electrophysiological consistency within and among large batches of manufactured cells?

In conclusion, we observed substantial variability in AP electrophysiology of hESC-CMs contained among and even within individual cardiac cell clusters, despite measures designed to reduce differences in experimental test conditions. The variability could not be correlated with beat rate, and therefore reflects genuine phenotypic differences. Our results suggest that in early stage differentiated cell clusters, a wide range of electrophysiological phenotypes is present, and in some clusters, one or two phenotypes can dominant. Finally, the use of high resolution optical mapping and AP analysis may be useful in the development of hESC-CMs for drug cardiotoxicity screening, disease modeling and myocardial repair.

## Methods

### Cardiac differentiation of human embryonic stem cells

The H9 line of human embryonic stem cells was used for this study. Spontaneous beating hEBs were obtained using a forced aggregation method as previously described^20^,^26^. Beating areas of hEBs from the same differentiation batch were mechanically dissected on day 15 of differentiation, and then transferred to plastic coverslips coated with 0.1% gelatin (Sigma-Aldrich, St. Louis, MO). These cardiac cell clusters were maintained in culture for at least 5 days to attach and recover spontaneous beating.

### Optical mapping

On day 21–24 of differentiation, cardiac cell clusters were stained with 10 μM voltage-sensitive dye di-4-ANEPPS (Invitrogen, Grand Island, NY) for 10 min, and then transferred to a custom-made mapping chamber with Tyrode’s solution containing 50 μM blebbistatin (Sigma-Aldrich, St. Louis, MO) to inhibit motion. Temperature was controlled at 37 °C throughout all experiments. For externally paced recordings, a pair of platinum field electrodes was used to deliver a 5–10 ms rectangular electrical stimulus. Pacing rate started at 60 beats per minute (bpm), and was incremented in steps of 30 bpm until the cluster failed to maintain 1:1 capture by the pacing stimulus.

Optical action potentials were recorded using a MiCAM Ultima-L CMOS camera (SciMedia, Costa Mesa, CA) with 100 × 100 pixels (16 μm/pixel) at 500 frames per second (fps). Sixteen to 32 seconds of recordings were taken for each cluster at each pacing rate.

### Signal processing and data analysis

To improve signal quality, optical recordings were convolved with a 5 × 5 boxcar filter. The border of each cluster was then traced manually to identify the recording pixels contained within that cluster. For paced activities, multiple APs from each recording site were temporally aligned by their activation times (calculated as the time of fastest AP upstroke), after which an average of the APs was calculated for that recording site. The averaged APs were quantified by 4 parameters: AP duration at 30% and 80% repolarization (APD_30_ and APD_80_, respectively), triangulation (time from APD_30_ to APD_80_) and fractional repolarization time (time from APD_30_ to APD_80_ divided by APD_80_). For triangulation, we used APD_80_, rather than APD_90_ as originally defined^42^, because in some cells the AP baseline had a slow repolarizing tail following the repolarization phase. These four parameters were used to quantify AP variability within and among clusters. Principal component analysis^52^ was used to determine the linear combination of AP parameters that accounted for the most variability within the dataset by orthogonal transformation. Levene’s test of equal variances was used to compare the variability in APD_80_ at spontaneous or paced conditions.

### Automated grouping

Action potentials were recorded from clusters paced at 90 bpm and separated into groups based on their action potential morphology, using a recently published automated algorithm^47^. This grouping algorithm had been developed using data from 9 of the 32 clusters presented here. Briefly, APs were divided into 2 or 3 groups based on an unsupervised, spectral clustering technique that amasses APs with similar shapes together and separates APs having different shapes. The similarity between pairs of APs is calculated mathematically as a weighted function based on the cumulative squared errors between the two temporal waveforms. Davies-Bouldin index (DBI)^53^ was also calculated using the cumulative squared errors, to compare cluster separation using different numbers of groups. Linear decision boundaries in pairs of AP parameter spaces were derived by support vector machines (SVMs) using labels determined by the spectral clustering.

## Additional Information

**How to cite this article**: Zhu, R. *et al.* Variability of Action Potentials Within and Among Cardiac Cell Clusters Derived from Human Embryonic Stem Cells. *Sci. Rep.* **6**, 18544; doi: 10.1038/srep18544 (2016).

## Figures and Tables

**Table 1:** Variability of AP parameters within individual and among all 32 clusters paced at 90 bpm.

	APD_30_	APD_80_	Triangulation	Fractional repolarization
Individual Mean	46 to 188 ms	85 to 257 ms	45 to 117 ms	0.27 to 0.51
Overall Mean	85 ms	137 ms	70 ms	0.41
Individual SD	4.3 to 30 ms	6.5 to 34 ms	4.8 to 58 ms	0.03 to 0.11
Overall SD	33 ms	41 ms	24 ms	0.09
